# Mentalization-based treatment for adolescents with conduct disorder (MBT-CD): a feasibility study

**DOI:** 10.1007/s00787-022-02113-4

**Published:** 2022-11-25

**Authors:** Sophie Hauschild, Lea Kasper, Jana Volkert, Esther Sobanski, Svenja Taubner

**Affiliations:** 1 Institute for Psychosocial Prevention, University Hospital Heidelberg, Heidelberg, Germany; 2 Psychological Institute, University of Heidelberg, Heidelberg, Germany; 3 Department of Psychology, Medical School Berlin, Berlin, Germany; 4 Department of Child and Adolescent Psychiatry and Psychotherapy, University Medical Center Johannes Gutenberg University Mainz, Mainz, Germany; 5 Department of Psychiatry and Psychotherapy, Medical Faculty Mannheim, Central Institute of Mental Health, University Hospital Heidelberg, Heidelberg, Germany; 6 Department of Child and Youth Psychiatry, Lucerne Psychiatry, Lucerne, Switzerland

**Keywords:** Mentalization-based treatment, Conduct disorder, Adolescents, Feasibility

## Abstract

**Abstract:**

Conduct disorder (CD) is a common psychiatric disorder in youth characterized by persisting norm-violating or aggressive behavior. Considering high individual and societal burden, feasible and effective psychotherapeutic treatment is desirable. Yet, treatments and research in this patient group are scarce. This study investigates the feasibility of mentalization-based treatment for adolescents with CD (MBT-CD) in terms of acceptability of MBT-CD and scientific assessments by participants as well as necessary organizational resources to conduct a consecutive randomized controlled trial (RCT). Recruitment, adherence and treatment session numbers were descriptively analyzed. Treatment evaluation interviews were qualitatively analyzed. A subset of sessions of therapists without prior MBT experience was rated for MBT adherence. Quantitative data were used to plan a consecutive RCT. Pre to post treatment changes in diagnosis and self-reported aggression, mentalizing and personality functioning were preliminarily analyzed. *N* = 45 adolescents with CD were recruited. 43% dropped out. Acceptance of scientific assessments was somewhat lower than therapy adherence (questionnaires filled out by ~ 80% of adolescents in treatment), and low at follow-up (25% of treatment completers). Mean session number was 30.3. Most treatment completers were satisfied with MBT-CD. Referrals mainly came from child and youth services and psychiatry. Nine of 16 sessions rated for MBT adherence were adherent. A priori sample size estimation for a prospective RCT with a drop-out rate of 43% yielded a sample of *N* = 158 to detect an effect *f* = .15 with 80% power in a repeated measures ANOVA. Pre–post analyses revealed diagnostic improvement in 68%. Of self-reported data, empathy pathology improved. Findings provide a sound basis for a consecutive feasibility and pilot RCT.

**Trial registration:**

Clinicaltrials.gov, registration number NCT02988453, November 30, 2016, https://clinicaltrials.gov/ct2/show/NCT02988453

## Introduction

Conduct disorder (CD) has been consistently identified as the second most common psychiatric disorder in youth [1, 2]. CD is defined as a repetitive and chronic pattern of aggressive behavior towards people, animals or other people’s property, norm-violating behavior and deceitfulness or theft (DSM-5; 3). Prevalence rates amongst children and adolescents range from 2 to 10% [1, 4] and are especially high amongst criminal offenders (~ 50%, [5]). The prognosis is often unfavorable [cmp. 6]. Thus, a feasible and effective treatment reducing behavior indicative for CD can be considered highly valuable with regard to individual and societal burden [7].

While psychological interventions have shown to be effective in treating youth with conduct problems (overview, e.g., in [8]), especially adolescents from middle adolescence and older are underrepresented in effective treatment programs (e.g., [9]). Treatment programs and scientific investigations for this group are still scarce (e.g., [10]). In Germany, a large-scale study on the diagnoses of youth receiving outpatient psychotherapy revealed that despite its prominent rank in prevalence in the general population [1], CD was only the seventh common psychiatric diagnosis in youth receiving outpatient psychotherapy [11]. Guideline recommendations favoring parent training over individual treatment in younger children might play a role in this divergency [11]. Yet, it seems plausible that other disorder-inherent aspects may impede treatment especially in older adolescents. First, adolescents’ externalizing behavior comes along with lack of agency and difficulties to see their own contribution to problems [10]. Moreover, adolescents strive for autonomy (e.g., [10]) and are assumed to have low epistemic trust [12], i.e., low trust that socially transmitted information is relevant and generalizable [13]. Consequently, going to therapy to work on themselves and get help from another might seem counterintuitive. Second, therapeutic pessimism caused by the long-lasting notion that antisocial individuals are untreatable [14] still might be a factor impeding treatment on the therapists’ side.

Thus, it is relevant that a psychological treatment can be perceived by adolescents as helpful in achieving their goals and interpersonally rewarding. Moreover, therapeutic pessimism needs to be revised and substituted with a treatment optimism, likely fostered by providing an etiological model to conduct problems in adolescence and model-based interventions to target them [14]. Such optimism seems warranted as adolescence is seen as a ‘window of opportunity’ due to high plasticity of the brain in this developmental phase and the chance to successfully take important developmental steps [15].

To reduce the treatment gap, mentalization-based treatment for adolescents with CD (MBT-CD) has been developed [16]. Mentalizing is the ability to reflect on mental states potentially underlying behavior [17]. Attachment disruptions and deficits in mentalizing are assumed to be one underlying cause of aggressive behavior and have repeatedly been demonstrated in individuals with conduct problems [18, 19]. Mentalizing deficits are described as an increased threat perception in social signals or a reduced sensitivity towards others’ distress [20], triggering aggressive behavior as a survival mechanism, or underlying a failure to inhibit aggressive behavior, respectively. The biobehavioral switch model [21] is used to describe the relationship between mentalizing and emotional arousal. Applying it to CD, Hauschild et al. [22] stressed the relevance of high emotional arousal by suggesting that the occurrence of CD symptomatology in a specific moment results from individuals frequently reaching their switch point in interpersonal interactions. According to the biobehavioral switch model [21], in a state of high emotional arousal, a change in mentalizing occurs via a transition from mainly cortically mediated, reflective processing of information to subcortically steered, automatic processing leading to fight and flight reactions. Neurobiological support for relevance of the switch in CD comes from investigating the neural response to interpersonal provocation in young violent offenders [23]: Their neural response was characterized by specific recruitment of areas in the brain stem fostering fight/flight reactions where non-violent individuals recruited areas related to the initiation of freezing. Moreover, deficits in parental mentalizing, which may be connected to disruptive attachment relationships, have been established as important for CD etiology; However, especially in older children and adolescents, deficits in parental mentalizing may serve more as a symptom maintaining factor [24]. In MBT-CD, therapists embrace the mentalizing stance of “not-knowing” about mental states underlying behavior as key to avoid patronizing in the face of destructive aggression. MBT-CD centers around gaining a psychological understanding of the adolescents’ aggression, enabling them to psychologically “buffer” their arousal by mentalizing emotions which elicit aggression. At the same time, adolescents experience being seen as an individual with important mental states steering their behavior which is assumed to enhance the adolescent’s feeling of agency and autonomy and reduce the need for CD symptomatology [16]. This study investigated the feasibility of MBT-CD in adolescents with a main diagnosis of CD or its milder variant oppositional defiant disorder (ODD). Feasibility was investigated in terms of acceptability of MBT-CD and scientific investigations by participants as well as necessary organizational resources to conduct a consecutive randomized controlled trial (RCT). Secondary aim was a pilot pre–post treatment investigation of change in CD or ODD diagnosis and self-reported aggression, mentalizing and personality functioning.

## Methods

### Design

This single-arm feasibility study was conducted between September 2016 and December 2021 at outpatient departments in Heidelberg (Institute for Psychosocial Prevention of the University Hospital Heidelberg, Centre for Psychosomatic Medicine) and Mainz (Rheinhessen Fachklinik, Department of Child and Adolescent Psychiatry and Psychotherapy of the University Medical Centre Johannes Gutenberg University Mainz), Germany. The study design and procedure are outlined in detail in the study protocol [25]. MBT-CD was delivered by therapists who were in or already finished psychotherapy training and who participated in an MBT-CD training conducted by the last author in Heidelberg (ST) and by the last and first author (ST & SH) in Mainz. Therapists in Heidelberg (*N* = 8, seven female) were trained in psychodynamic therapy and had a mean age of 35.1 (*SD* = 8.1). Therapists in Mainz (*N* = 6, all female) were trained in cognitive behavior therapy (CBT; *N* = 4) and psychodynamic therapy (PT; *N* = 2) and had a mean age of 31.7 years (*SD* = 4.8). Sessions were videotaped. Supervision was provided by the last author (ST) biweekly to monthly and supported by the first and third authors (SH & JV).

The study first started as an RCT to investigate the effectiveness of MBT-CD compared to treatment as usual (TAU) and was changed in consent with the funders into a single-arm feasibility and pilot study due to recruitment and randomization difficulties in the first two years of the study. The design of the feasibility study was adaptive, i.e., changes in intervention and scientific assessments were made over the course of the study to improve treatment and study retention on the basis of patient experience.

### Study participants

Adolescents between eleven and 18 years with a main CD or ODD diagnosis were offered MBT-CD for six to 12 months. Exclusion criteria were having committed sexual offenses, acute psychotic symptoms, early-onset schizophrenia, neurological or intelligence impairments, non-German-speaking or other clinical contraindications for outpatient psychotherapy (e.g., acute suicidality). Participants were recruited at the participating centers, via leaflets and personal or telephone contact with multipliers and institutions (e.g., child and youth welfare services, schools, police stations, probation officers).

**Fig. 1:**
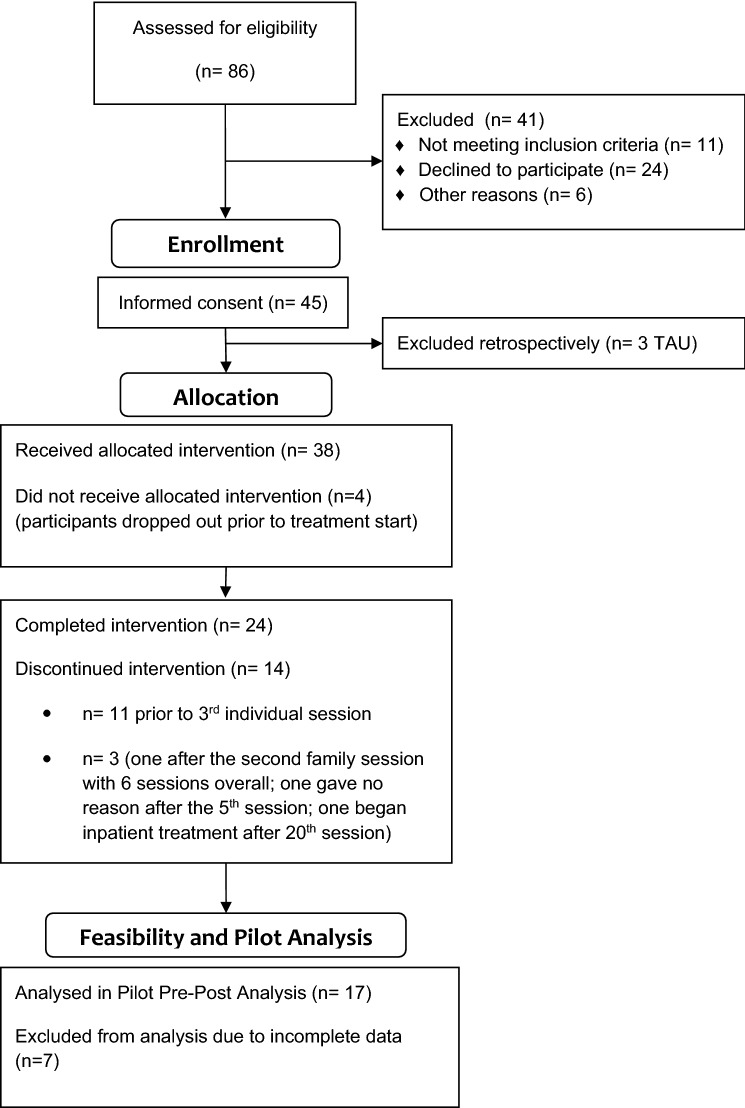
Consort flow diagram of trial phases from screening to pilot analysis

Participants were screened for eligibility (either directly or indirectly through caretaker report) through a standardized checklist assessing conduct problems. At the beginning (T1) and end of treatment (T3), diagnostic assessments were conducted including the CD and ODD sections of the MINI Kid [26] and SCID II [3]. Moreover, participants were asked to fill out questionnaires at four timepoints: at T1, during treatment (T2), at T3 and three months after the end of treatment (follow-up, T4). Questionnaires measured adolescents’ experience of trauma, psychopathy (both only at baseline), aggressive behavior, mentalizing, personality functioning, parental behavior, avoidant or anxious attachment and the therapeutic relationship. Parents were asked to assess their stress of being the parent of an adolescent, their mentalizing and the therapeutic relationship as well. Adolescents received a total of 50€ for taking part in the scientific assessments. An overview of study flow and measures can be found in the study protocol by Taubner et al. [25] (Fig. 1). Both adolescents and their parents gave written informed consent before participating. The study was approved by the Ethics Committee of the Heidelberg University Medical Faculty (Germany; S-534/2016) and registered at clinicaltrials.gov (NCT02988453).

### Intervention

MBT-CD included one weekly individual session and one monthly family (or other caretakers) session over the course of 6 to 12 months. Flexibility in duration and number of individual or family sessions was exhibited throughout the study to provide optimal care for each patient. MBT-CD started with two psychoeducational sessions for the adolescent and their family on mentalizing and reciprocal effects of difficulties with mentalizing and handling emotionally challenging situations. In individual and family sessions, MBT-CD targeted recovery or establishment of mentalizing in emotionally challenging situations for the adolescents. During the first individual sessions, the adolescent’s problem behavior was mentalized using a Motivational Interviewing technique [27] to investigate pros and cons as well as subjective confidence and relevance for change. Concomitantly, mentalizing resources and difficulties were diagnosed and written down in a case formulation in form of a letter to the patient. Together with the adolescent, the case formulation was worked through, changed where appropriate and agreed upon as therapy focus. In the following, therapists managed sessions around the focus. After the end of treatment, monthly booster sessions were offered to stabilize mentalizing achievements. Therapists worked collaboratively with youth welfare services, if these were involved.

### Self-report measures

Aggression was measured with the 32-item Subtypes of Antisocial Behavior Questionnaire [STAB; 28], with the subscales physical, social and rule-breaking aggression. The 23-item Reactive-Proactive Aggression Questionnaire (RPQ) was used to measure reactive and proactive aggression [29]. Personality Functioning was measured with the Levels of Personality Functioning Questionnaire 12–18 for adolescents [LoPF; 30]. Mentalizing was measured with the Reflective Functioning Questionnaire [RFQ; 31], analyzed following recommendations by Müller et al. [32]. For all measures, reliability and validity has been demonstrated [28–32]. Internal consistency was at least acceptable for all scales in the current study (all Cronbach’s alphas > 0.76), except for the STAB subscale social aggression at T3 (Cronbach’s alpha = 0.68).

### Analysis

To investigate the acceptability of MBT-CD and scientific assessments, recruitment and therapy adherence rates as well as mean, standard deviation and range of attended session numbers in completed treatments were calculated. Moreover, oral evaluations of treatment and scientific assessments from treatment completers assessed via semi-structured interviews adapted from Krause et al. [33] were qualitatively analyzed to identify hindering and helpful aspects from the patient’s point of view.

To investigate necessary organizational resources to conduct an MBT-CD RCT, recruitment numbers were descriptively investigated with regard to adaptations made throughout the study, study center characteristics and referring institutions. Additionally, to gain insight into the success of MBT trainings and supervision for MBT implementation, the MBT adherence and competence scale [MBT-ACS, 17, https://www.annafreud.org/training/mentalization-based-treatment-adults-old/mbt-adherence-scale/] was applied by the first author on a subset of sessions conducted by therapists without prior MBT experience. Adherence of therapies conducted by therapists with advanced (SH, LK) to expert (ST) MBT experience (38% of completed therapies) was beyond the scope of this study. The MBT-ACS allows the assessment of (in)adequate use of key MBT elements on domains throughout the session (e.g., not-knowing stance) and when indicated (e.g., addressing non-mentalizing). Ratings range from one to seven, with four representing a “good enough” use of MBT intervention. An overall session score below 3.5 indicates a non-adherent session. An authorized translation of the scale into German had been conducted by our workgroup beforehand. It has to be noted that, while the first author has years of experience in MBT research and practice, reliability of ratings was not tested within this study. Thus, ratings represent a first clinical impression of model adherence in therapists without prior MBT experience.

For video selection, only completed therapies were chosen, ensuring that supervision for therapy was provided for several hours. For those therapists who had more than one completed therapy, the one therapists subjectively believed was most on the model was selected. For each selected therapy, two sessions were randomly chosen (one in the first and one in the second half of therapy). In Heidelberg, the criteria resulted in four therapies. In Mainz, out of five therapists fulfilling these criteria, only the four with a CBT orientation (another had a psychodynamic orientation) were chosen for reasons of homogeneity within centers. Thus overall, 16 sessions were rated for MBT adherence.

Moreover, an a priori sample size estimation was conducted with G*Power and data on recruitment and drop-out rates were used for estimation of a prospective RCT’s exemplary study duration and necessary number of cooperating centers.

For the preliminary pilot pre–post analysis, complete data on core measures (adolescents’ aggression, mentalizing, personality functioning) were used (available for *N* = 17 adolescents) and the 95% confidence intervals of the mean pre to post differences were calculated in R. Missing questionnaire values up to 20% were imputed with single person mean imputation on the item level.

## Results

### Description of the recruited sample

Forty-five adolescents agreed to participate, 29 (64%) of those in Heidelberg and 16 (36%) in Mainz. Thirty-four (76%) were diagnosed with CD, eleven (24%) with ODD. Thirty (67%) identified as male, 15 (33%) as female. Mean age was 14.4 (*SD* = 2.1; range: 11 – 18). Eleven adolescents (24%) went to lower secondary school (German: “Haupt-, Werkrealschule”), 13 (29%) to higher secondary school (German: Realschule), seven (16%) to high school, (four (9%) to special needs school, one (2%) to vocational school. For six adolescents (13%) differentiation was not possible (comprehensive school), and three (7%) did not go to school at intake.

### Acceptability of intervention and scientific investigation in patient group

#### Recruitment and therapy adherence

Overall, 86 adolescents were screened for eligibility across an overall study duration of five years and four months. Of these 86, 41 (48%) could not be included: 24 (28%) declined participation in the screening process, eleven (13%) were excluded in the screening process because there was no indication. Six (7%) were excluded due to other reasons (e.g., postponement of appointments by the adolescent until recruitment stop). Forty-five adolescents (52% of screened individuals) agreed to participate in the study. Three (3%) participants were randomized and referred to TAU before design change of the study; they were defined as study determined drop-out and are, thus, not considered for calculation of therapy adherence, acceptance of scientific assessments and pre–post analyses.

Of the remaining 42, four (10%) dropped out between diagnostic process and beginning of the intervention. Twenty-four (57%) completed the treatment. 14 (33%) dropped out during the intervention (for overview see flow diagram Fig. 1). Of these, eleven (79%), dropped out prior to or after the second individual session with their therapist.

#### Acceptance of scientific assessments

Acceptance of scientific assessments at a given timepoint was defined to be present when participants filled out at least one questionnaire at the respective timepoint. The number of adolescents and parents filling out at least one questionnaire is presented in ratio to the number of adolescents still participating in the study at the given timepoint: At T1 pre-treatment, questionnaires were filled out by 32 adolescents (84% of 38 treatment beginners) and 31 parents (82%). At T2 during treatment, questionnaires were filled out by 19 adolescents (76% of 25 still participating adolescents) and 13 parents (52%). At T3, questionnaires were filled out by 20 adolescents (83% of 24 completers) and 13 parents (54%). At T4, 6 adolescents (25% of the 24 completers) and 5 parents (21%) filled out the online questionnaires.

#### Treatment duration

Mean session number of completed treatments was 30.3 (*SD* = 15.2), with a range of eight to 69 individual sessions and zero to ten family sessions. Lowest numbers of individual sessions per treatment were eight (treatment duration: eight months), ten (treatment duration: seven months) and 12 (treatment duration: seven months). Highest numbers of individual sessions were 50 (treatment duration: 20 months), 56 (treatment duration: 23 months), and 69 (treatment duration: 26 months).

#### Qualitative treatment evaluations

Overall, 16 interview-based treatment evaluations were available for qualitative analysis (*N* = 10 in Heidelberg, *N* = 6 in Mainz). A detailed content analysis of treatment evaluations of the Heidelberg subsample published as a sub-analysis [22] revealed several positive aspects: adolescents liked, that they had someone who listened to and understood them. Therapy was deemed helpful for problem-solving and reflection. Mentalizing processes were named most often as important moments in therapy (e.g., gaining more self-control with increasing insight into inner states). While some adolescents indicated that their symptoms improved through therapy, some did not report changes through therapy, or attributed change to life-events or personal development. For some, the term mentalizing had some meaning (e.g., “understanding the other person’s emotions and behaviors and reacting correspondingly”). The psychoeducation was evaluated positively by half of the adolescents due to active involvement of their family or themselves; however, most did not remember the content. As negative aspects, adolescents most often reported that their therapists asked “too many questions”, which was “annoying”. Moreover, critique concerning the less structured phases of treatment was uttered especially by younger patients [22].

The six therapy evaluations from Mainz revealed a similar picture: Three reported they were satisfied with the treatment because it helped them fight less with their families, increase their insight into other people’s feelings, and reduce the urge to fight instead of seeking a solution for problems (e.g., “I learned to tell my friends I need a short break to calm down instead of quitting on them. This helped me not to lose them.”). Three indicated that they changed due to life-events and not therapy. Positive aspects included that therapists were more easy-going than therapists before, and that they could choose for themselves what they wanted to talk about rather than being told what to do. Negative aspects concerned the setting (having to come once a week) and “boring” diagnostic procedure. Similar to the Heidelberg sample, two adolescents did not remember the psychoeducation, and two indicated that they found it neither good nor bad. Three defined the word mentalizing as taking others’ perspectives; one said it was additionally about thinking about one’s own feelings. Most adolescents of both centers thought the scientific assessments were too long and they were annoyed by being asked “the same things twice”.

### Organizational requirements

#### Recruitment potential per center, difficulties and study adaptations

In Heidelberg, 55 individuals were screened within 36 months of recruitment. Of those, eight did not fulfill participation criteria. Of 47 individuals potentially eligible, 29 (62%) were recruited for study participation. The mean overall recruitment rate per month was 0.9. Yet, monthly recruitment rates increased over the course of the study: During the first 24 months, 14 adolescents were recruited, i.e., recruitment rate was around 0.6 per month. At the time, parts of the diagnostic appointments were located in the child and youth psychiatry and part of it in the institute where the study was conducted. As a result, concurring studies were offered at the child and youth psychiatry, and changes in personnel and location seemed to confuse prospective participants. Moreover, participants recruited by the study institute reported reservations towards the child and youth psychiatry; and those recruited by the child and youth psychiatry reported reservations towards the study program as it was unfamiliar. Recruitment rates were lower than necessary for successful implementation of the RCT design. Consequently, the design was changed into a single-arm feasibility study, diagnostic appointments did not any longer include visits to the child and youth psychiatry and recruitment was stimulated again. In the third and last recruitment year in Heidelberg, 15 adolescents were recruited, i.e., the recruitment rate was doubled to around 1.3 per month.

In Mainz, 31 adolescents were screened within 16 months of recruitment. Of those, three adolescents did not fulfill participation criteria. Of 28 individuals potentially eligible for study participation, 16 (57%) were recruited for participation. The mean overall recruitment rate per month was one. Therapies in Mainz were conducted in the outpatient department of the child and adolescent psychiatry, so that in contrast to the Heidelberg study center, recruitment numbers likely benefitted from close connection to the child and adolescent psychiatry. Notably, eleven of the 16 recruited adolescents were included in the first seven months of recruitment from September 2019 to March 2020 up until the beginning of the first wave of the COVID-19-pandemic. In the last five months of recruitment from August to December 2020, five more adolescents were recruited, while from April to July 2020 none were included into the study. Thus, the number of potential participants in Mainz was likely reduced by the start of the pandemic in the middle of the recruitment phase.

#### Recruitment networks

Especially with study centers not directly connected to recruitment of a clinic, recruitment depended on a network. For the 29 participating adolescents in Heidelberg, nine (31%) were referred from community child services. For three (33%) of those, treatment was court ordered. Seven (24%) were referred from the child and youth psychiatry/general psychiatry. Three (10%) were referred from schools, three (10%) from therapists. Two (7%) came from youth centers or youth homes. One (3%) was referred from a health insurance company and one (3%) from peers familiar with the program. For three (10%) adolescents referral was not documented (recruited before design change). Of the 16 adolescents in Mainz, four were recruited from the inpatient (25%), eleven (69%) from the outpatient department of the child and adolescent psychiatry and one (6%) from an already participating sibling.

#### MBT adherence of therapists without prior MBT experience

Mean adherence of the 16 sessions was 4 (*SD* = 0.9), with scores between 2.8 and 5.4, i.e., in part below the threshold of being adherent (3.5, [17]). Nine (56%) of the 16 sessions were adherent. Three sessions (19%, conducted in Heidelberg) did not fulfill the “knock-out” criterion of the therapist displaying the not-knowing stance throughout the session. They could therefore not be labeled as MBT sessions. Four (25%) with scores below 3.5 were characterized by a strong focus on exploring behavior instead of mental states while still displaying great interpersonal warmth and developing the relationship (*N* = 2, 13%, conducted in Mainz), too little affect focus with MBT interventions not meeting the patient (*N* = 1, 6%, conducted in Mainz), and cognitive re-structuring coming along with a shortcoming in addressing non-mentalizing (*N* = 1, 6%, conducted in Mainz).

#### Organizational requirements for planning of a prospective RCT

An a priori sample size calculation was carried out in G*Power for a 2 (MBT-CD vs. control intervention) × 2 (pre vs. post treatment) mixed design analyzed with a repeated measures analysis of variance. Effect of interest was the between–within interaction effect; a small to medium effect size of *f* = 0.15 was expected [cmp. 8, 34]. Alpha level was set to 0.05, power to 0.80 and correlation amongst repeated measures to r = 0.5. The calculation resulted in a total sample size of *N* = 90. Calculated with 43% of drop-out observed in our study, 158 participants would be needed. Assuming a recruitment rate of 1.3 participant per month per, and a recruitment period of ~ 30 months, an effectiveness trial comparing MBT-CD with a control intervention would require at least four recruitment centers. Importantly, this calculation is based on the mean recruitment rate in Heidelberg when a recruitment network had already been established. When centers without an established recruitment network are included, a lower monthly recruitment rate should be considered.

### Pilot pre–post analysis

#### Conduct disorder and oppositional defiant disorder diagnoses

At the end of treatment, 22 (92%) of 24 treatment completers attended a post treatment diagnostic session. Of those 22, 15 (68%) improved with regard to their diagnoses: 13 (59%) adolescents did not fulfill CD or ODD criteria anymore; for two (9%) adolescents, diagnosis was improved from CD to ODD. Seven (32%) still fulfilled criteria of CD or ODD, respectively (six with CD and one with ODD at T1).

#### Questionnaire data

Due to incomplete data and low sample size, questionnaire data were only drawn to for a pilot pre–post analysis. For 17 adolescents, complete data for the core measures at t1 and t3 were available. 95% confidence intervals of mean differences between pre- and post-treatment data did not indicate significant pre to post changes in aggression, mentalizing and personality pathology except for empathy pathology: The confidence interval of the mean difference between empathy pathology at t1 and t3 indicated a significant improvement of empathy (mean difference: 6.3, *SD* = 11.3, 95% CI [0.5; 12.2]; for overview of means, standard deviations, lower and upper bounds of the confidence intervals of mean differences for the whole sample and for each center, respectively, see Tables 1 and 2).

**Table 1:** Means (AM), standard deviations (SD) and the 95%-Confidence Interval (CI) of the pre to post differences of aggression, personality functioning and mentalizing of *N* = 17 adolescents

Scale (Measure)	T1 (AM, SD)	T3 (AM, SD)	Difference (AM, SD)	95% CI of Difference
Physical aggression (STAB)	28.1 (9.3)	24.5 (9.8)	3.5 (10.6)	[− 1.9; 9.0]
Social aggression (STAB)	23.8 (7.3)	19.9 (4.7)	3.9 (8.2)	[− 0.3; 8.9]
Rulebreaking (STAB)	18.1 (8.1)	18.4 (8.0)	− 0.3 (7.8)	[− 4.3; 3.7]
Total aggression (RPQ)	13.6 (6.8)	11.5 (7.3)	2.2 (8.6)	[− 2.2; 6.6]
Reactive aggression (RPQ)	10.0 (4.4)	8.4 (4.4)	1.6 (5.4)	[− 1.2; 4.4]
Proactive aggression (RPQ)	3.6 (3.5)	3.1 (3.6)	0.6 (3.8)	[− 1.4; 2.5]
Identity pathology (LoPF)	31.0 (15.4)	29.9 (11.1)	1.1 (15.0)	[− 6.7; 8.8]
Steering pathology (LoPF)	34.1 (17.9)	28.8 (19.5)	5.3 (20.0)	[− 5.0; 15.6]
Empathy pathology (LoPF)	36.6 (15.5)	30.3 (14.0)	6.3 (11.3)	[0.5; 12.2]
Intimacy pathology (LoPF)	26.4 (13.3)	28.3 (13.7)	− 1.9 (12.7)	[− 8.5; 4.6]
Mentalizing Uncertainty (RFQ)	3.4 (1.2)	3.3 (1.5)	0.2 (1.5)	[− 0.6; 0.9]

*STAB* Subtypes of Antisocial Behavior Questionnaire [28], *RPQ* Reactive–Proactive Aggression Questionnaire [29], *LoPF* Levels of Personality Functioning Questionnaire 12–18 [30], *RFQ* Reflective Functioning Questionnaire [31], analyzed following recommendations by Müller et al. [32]

**Table 2:** Means (AM), standard deviations (SD) and the 95%-Confidence Interval (CI) of the pre to post differences of aggression, personality functioning and mentalizing of *N* = 8 adolescents in Heidelberg (upper half) and *N* = 9 adolescents in Mainz (lower half)

Scale (measure)	T1 (AM, SD)	T3 (AM, SD)	Difference (AM, SD)	95% CI of Difference
Heidelberg sample (*N* = 8)				
Physical aggression (STAB)	27.6 (10.8)	21.9 (9.9)	5.8 (4.6)	[1.9; 9.6]
Social aggression (STAB)	27.5 (8.4)	20.4 (4.3)	7.1 (7.6)	[0.8; 13.5]
Rulebreaking (STAB)	19.1 (9.9)	16.6 (6.6)	2.5 (3.4)	[− 0.3; 5.3]
Total aggression (RPQ)	14.3 (8.1)	10.3 (7.0)	4.0 (6.9)	[− 1.8; 9.8]
Reactive aggression (RPQ)	10.0 (4.8)	7.4 (3.7)	2.6 (5.2)	[− 1.8; 7.0]
Proactive aggression (RPQ)	4.3 (4.2)	2.9 (4.1)	1.4 (4.7)	[− 0.7; 3.4]
Identity pathology (LoPF)	32.6 (20.2)	31.6 (14.5)	1.0 (19.3)	[− 15.1; 17.1]
Steering pathology (LoPF)	39.4 (21.1)	32.9 (19.4)	6.5 (9.2)	[− 1.2; 14.2]
Empathy pathology (LoPF)	38.7 (17.1)	30.8 (15.7)	8.0 (9.4)	[0.1; 15.8]
Intimacy pathology (LoPF)	25.9 (15.2)	29.3 (15.3)	− 3.4 (9.5)	[− 11.3; 4.5]
Mentalizing Uncertainty (RFQ)	3.5 (1.5)	3.5 (1.3)	0.0 (1.2)	[− 1.0; 1.0]
Mainz sample (*N* = 9)				
Physical aggression (STAB)	28.4 (8.4)	26.9 (9.6)	1.6 (14.1)	[− 9.3; 12.4]
Social aggression (STAB)	20.4 (4.5)	19.4 (5.2)	1.0 (8.0)	[− 5.1; 7.1]
Rulebreaking (STAB)	17.2 (6.6)	20.0 (9.2)	− 2.8 (9.9)	[− 10.4; 4.8]
Total aggression (RPQ)	13.1 (5.9)	12.6 (7.7)	0.6 (9.9)	[− 7.1; 8.2]
Reactive aggression (RPQ)	10.0 (4.3)	9.3 (5.0)	0.7 (5.6)	[− 3.7; 5.0]
Proactive aggression (RPQ)	3.1 (2.9)	3.2 (3.5)	− 0.1 (4.7)	[− 3.7; 3.5]
Identity pathology (LoPF)	29.6 (10.7)	28.4 (7.1)	1.1 (11.2)	[− 7.5; 9.7]
Steering pathology (LoPF)	29.3 (14.2)	25.1 (20.0)	4.2 (26.8)	[− 16.4; 24.9]
Empathy pathology (LoPF)	34.8 (14.7)	29.9 (13.4)	4.9 (13.2)	[− 5.2; 15.0]
Intimacy pathology (LoPF)	26.8 (12.3)	27.4 (13.0)	− 0.7 (15.5)	[− 12.6; 11.3]
Mentalizing Uncertainty (RFQ)	3.4 (0.8)	3.1 (1.6)	0.2 (1.8)	[− 1.1; 1.7]

*STAB* Subtypes of Antisocial Behavior Questionnaire [28], *RPQ* Reactive–Proactive Aggression Questionnaire [29], *LoPF* Levels of Personality Functioning Questionnaire 12–18 [30], *RFQ* Reflective Functioning Questionnaire [31], analyzed following recommendations by Müller et al. [32]

## Discussion

This study investigated the feasibility of MBT-CD for adolescents in terms of acceptability of intervention and scientific assessments by participants as well as necessary organizational resources. The following aspects supporting feasibility were identified: First, adolescents with CD could be recruited for participation. Second, once adolescents stayed beyond session two, they completed treatment with high probability. However, recruitment was slow, especially when recruitment networks were not yet established. Moreover, follow-up acceptance of scientific assessments was low. In line with the well-known difficulty to reach this group (e.g., [10]), collaboration with several treatment centers which can already access recruitment networks is recommended for future trials to reach a sample size large enough to detect treatment effect with sufficient power. Follow-up assessments might need an extra personal appointment and additional financial reimbursement.

The screening–recruitment ratio of ~ 2:1 and low drop-out rates after the third individual session point to high relevance of very first contacts with adolescents with CD for therapy adherence. Several aspects characteristic for the sample of this study have previously been identified as risk factors for drop-out of adolescent psychotherapy as e.g., conduct problems, youth in middle adolescence [34, 35]) and being referred from others [34]. Yet, drop-out rates of adolescents who started MBT-CD were well within the range of drop-out of child and adolescent outpatient treatments: De Haan and colleagues [36] showed in their meta-analytic review on drop-out of various child and adolescent outpatient treatments a drop-out range from 28 to 75%. More recently, similar rates of 37% drop-out of adolescent therapy for depression [35] and 45% drop-out of mentalization-based group therapy for adolescents with borderline pathology [37] were observed. Thus, future studies should investigate how patient characteristics, as, e.g., the presence of callous-unemotional traits [3], and the therapeutic relationship might influence therapy adherence in adolescents with CD.

Moreover, feasibility seems likely to be fostered by several center characteristics: Whenever possible, treatment should be offered at the location of the first contact with the adolescents. Scientific assessments should optimally be short and conducted in a familiar environment, e.g., by their therapist or directly before or after treatment sessions by a researcher.

It seems to be partly feasible to teach MBT-CD to therapists of different therapeutic backgrounds without prior MBT experience by conducting a one- or two-day training and regular supervision. Yet, as not all therapists were adherent, one may speculate whether different therapeutic orientations came along with specific difficulties for example with taking the not-knowing mentalizing stance or exploring underlying mental states rather than behavior. Moreover, a lack of MBT adherence might result from the “contagious” potential of a patient’s non-mentalizing [38]. Addressing non-mentalizing adherently may take a considerable amount of practice. Thus, attending regular supervision with video- or audio-tapes of sessions might be crucial. As, however, learning MBT may present specific challenges (cf. also [39]), MBT trainings may additionally benefit from, e.g., mediational approaches [40]. Yet per therapist, only two sessions were rated by only one rater, so that interpretation is restricted.

The following treatment aspects might be considered in future trials: Both patient and study center characteristics seem important for the observed range in treatment duration. Treatments with low session numbers were conducted in Mainz with younger adolescents (< = 14 years). Treatments were characterized by inconsistent attendance of both, adolescents and their parents. High session numbers were conducted in Heidelberg with older adolescents (> = 16 years) and treatments were characterized by high complexity of symptom etiology, high psychopathy and/or legal complexity. The overall study treatment phase in Mainz was shorter than in Heidelberg (18 vs. 36 months). Additionally, PT orientation of therapists in Heidelberg and predominant CBT orientation in Mainz might have contributed to longer and shorter treatment durations, respectively. Based on these findings, MBT-CD might need to be adapted depending on the adolescents’ age or maturity: for adolescents aged 14 or younger, the first and highly structured phase of MBT-CD in combination with frequent family sessions may suffice. For adolescents 15 years or older, individual sessions might play a greater role with a focus on etiology of their aggression and negative (legal) consequences of their behavior. This is supported by implications drawn from results of the structured content analysis of the Heidelberg subsample: younger individuals seemed to prefer and benefit more from the more structured phase of the intervention, while older individuals may have benefitted more from the mentalizing processes unfolding over the course of their therapy [22].

One patient reported deterioration of symptoms in that she felt more depressed. With respect to future studies, this is a concerning albeit plausible outcome, as reduction of aggression might not uncommonly be succeeded by depressive symptoms. Thus, depressive symptomatology needs to be monitored throughout the study and cared for, respectively. This is also true for frequent comorbid substance use disorder. While substance use is not an exclusion criterion for MBT-CD, it should be focused on from the beginning and patients may need inpatient treatment for substance abuse prior to MBT-CD.

Potential center effects became apparent in the preliminary pre–post analyses as well as in the treatment evaluations by the adolescents: In Heidelberg, self-reported empathy pathology and aggression were reduced while this was not evident in the Mainz sample. Regarding treatment evaluations, adolescents in Heidelberg indicated they were annoyed by therapists’ questions, while adolescents in Mainz explicitly valued the non-patronizing stance of their therapists. While both aspects likely represent the mentalizing stance, the centers may have differed in their focus on adopting aspects of the stance, resulting in different perceptions by adolescents. However, due to the small sample size and lack of controlled investigation of skilful MBT implementation in the centers, these aspects need further investigation.

### Limitations and future directions

One major limitation of this study is that randomization and control intervention feasibility could not be investigated. Thus, results cannot be generalized to feasibility of an RCT in this patient group; these aspects should be addressed in future studies and treatment effects piloted. Researchers might consider broadening inclusion criteria in taking a more dimensional approach to personality pathology, which seems sensible considering the often chronic and progredient course of the disorder (cmp. [6]).

A second major limitation is the small sample size. While in line with the model, pre-to-post treatment improvements in diagnoses and empathy pathology were observed, small sample size and lack of control group preclude conclusions about possible treatment effects. For future studies, larger sample sizes must be achieved to ensure statistically sound evaluation of effects. Furthermore, alternative research designs may also be considered, such as cohort designs which have been discussed to better fit the investigation of psychotherapy effects in individuals with complex disorders [41].

Parents only inconsistently took part in the scientific assessments. This might be a result of the recommended flexibility in the frequency of family sessions. Yet moreover, it seemed to be indicative for parent ambivalences towards treatment. Active engagement of parents will need to be a goal just as much as of adolescents when aiming for high acceptance of scientific assessments from the parents.

Overall, the study provides a sound basis for conducting a consecutive feasibility and pilot RCT and relevant information for conducting a definitive RCT. Active relational engagement seems to be key during very first contacts with the patients. MBT-CD aims at helping therapists in gaining confidence to work with adolescents with CD and taking a non-judgmental, curious stance about their mental states. This aims at allowing adolescents to perceive themselves and others as feeling and thinking human beings and (re)gain control over their aggressive behavior.

## Data Availability

Anonymous data can be made available from the first author per request.
